# Fetal Androgens and Human Sexual Orientation: Searching for the Elusive Link

**DOI:** 10.1007/s10508-017-1021-6

**Published:** 2017-06-28

**Authors:** Vickie Pasterski

**Affiliations:** Department of Psychology, University of Cambridge, Free School Lane, Cambridge, CB2 3RQ UK

In the article targeted by this Commentary, Breedlove (2017) chronicles the progression of his thinking about hormonal influences on human behavior, including sexual orientation, across the span of his career. He considers the question in terms of expectations, based on what has been known at various points in time, versus the accumulation of data to present day. He broadly frames his argument within the commonly applied architecture of pre- versus postnatal factors, specifically considering prenatal androgen exposure and postnatal socialization influences. A critical distinction which quickly becomes evident is that potential mechanisms underlying the development of human sexual orientation, regardless of theoretical framework, likely differ for men and women. In this regard, Breedlove reaches conclusions that differ by sex: fetal androgen exposure contributes to sexual attractions in females while no such evidence exists for men. In this Commentary, I reconsider some of the same evidence and present further evidence for a revised interpretation of the extant literature.

With respect to ultimate conclusions in the target article, Breedlove explores several lines of evidence relevant to sexual orientation in the context of fetal hormonal exposure and social learning theories. Among these, he considers: (1) findings from studies of sexual orientation in individuals exposed to atypical levels of prenatal androgens, i.e., congenital adrenal hyperplasia (CAH; Pasterski & Hughes, 2016) and androgen insensitivity syndrome (AIS; Hughes et al., 2012); (2) the reliability and usefulness of specific physiological traits, i.e., otoacoustic emissions and 2nd to 4th finger–length ratios, as retrospective biomarkers of fetal androgen exposure with potential for studies of sex development; and (3) findings from studies using these retrospective biomarkers to assess potential influences of fetal androgen exposure on human sexual orientation. Evidence for potential mechanisms not discussed in the target article and that bear additional consideration include findings from longitudinal studies and investigations looking at the effects of androgens in the early postnatal period, i.e., mini-puberty. I will consider each of these in turn.

## Prenatal Hormones and Postnatal Socialization in CAH

Based on the organizational hypothesis (Phoenix, Goy, Gerall, & Young, 1959), hundreds of studies have established a role for fetal androgens in sexually dimorphic neurobehavioral development observed both in humans and in nonhuman vertebrates (Hines, 2010; Morris, Jordan, & Breedlove, 2004). While the animal literature is replete with experimental evidence of the potential to directly manipulate sexually dimorphic behavior, in both males and females, by changing exposure to androgens during critical periods of neural development, evidence in humans remains quasi-experimental due to ethical constraints. In humans, potentially confounding factors cannot be controlled. In considering the bulk of evidence that masculinized patterns of behavior occur in girls exposed prenatally to excess androgens due to CAH (Hines, 2011; Meyer-Bahlburg, Dolezal, Baker, & New, 2008), Breedlove acknowledges the potentially confounding factor that these girls are also born physically virilized. Masculinized behavior, including development toward a homosexual sexual orientation, could be due to socialization by parents affected by the appearance of the child at birth. Even so, understanding the degree of relative influences requires a nuanced understanding of such mechanisms. As Breedlove rightly points out, the majority of women with CAH are not lesbian. He fails, however, to integrate two important pieces of evidence that inform the interpretation and integration of the body of findings.

The first comes from a study which directly assessed parental socialization of children with and without CAH (Pasterski et al., 2005). We found that while parents showed expected reinforcement of sex-typical toy play, parents of girls with CAH showed increased reinforcement for play with girls’ toys compared to parents of unaffected girls. Furthermore, there was a significant inverse relationship between level of sex-typed reinforcement by parents and the time their daughters with CAH spent playing with girls’ toys. This suggests that the more these girls played with boys’ toys, or ignored the girls’ toys, the more the parents encouraged girl-typical play. Alternatively, greater encouragement by parents was met with greater resistance. Either way, there was no evidence of socialization toward a masculine presentation. Social desirability was ruled out as a possible explanation as well, as parents were not explicitly made aware of their own behavior in this study of childhood sex-typed behavior.

Effects of parental socialization influences notwithstanding, we know from the social learning literature that peers and other models are also sources of influence in the development of sex-typed behavior (Pasterski, Golombok, & Hines, 2011b). Further clarification on the intersection of hormonal influences and social learning comes from a study assessing imitation of same-sex models and responsiveness to explicit sex-typed labeling in children with and without CAH (Hines et al., 2016). Children were exposed to modeling of sex-typed preferences by adults, where males and females demonstrated sex-differentiated preferences for neutral objects, and to explicit labeling of neutral items as “for boys” or “for girls.” While unaffected boys and girls showed the expected effects of imitation and learning from gendered labels, girls with CAH showed reduced imitation of female models and reduced responsiveness to information that particular objects were for girls. Implications for the role of increased fetal androgen exposure in the context of subsequent social learning experiences are important. Even in the face of explicit socialization influences, girls with CAH did not engage in the self-socialization typical of unaffected children. Once again, however, we must consider that most women with CAH are not lesbian, and masculinization of other sex-typed behaviors, such as those described above, is not “complete.” That is, though the behavior of girls with CAH is more masculine than that of unaffected girls, they generally are not as masculine as typical boys (Pasterski et al., 2005, 2007, 2011a). Finally, though sexual function may be impaired in women with CAH, especially in cases of repeated surgical interventions to correct genital anomalies (Callens et al., 2012), fundamental physiological arousal patterns are likely not influenced to the point of reversal to same-sex attractions. Taken together, these studies suggest that sex-related development is even more nuanced and complex than previously considered in the framework of prenatal hormones versus postnatal socialization.

## Normative Development: Longitudinal Data, Biomarkers, and Twins

Studies of variability in fetal androgen exposure in normative populations provide another line of evidence for influences on sex-typed behavior, including sexual orientation. To that end, investigations are limited to intensive longitudinal studies beginning with measurement of fetal androgens in pregnancy and following offspring into (early) adulthood, or on retrospective biomarkers of early androgen exposure. On the former point, there are reports from the AVON Longitudinal Study of Parents and Children (ALSPAC; Golding, 2001), which include maternal blood testosterone measurements and longitudinal follow-up of children who are now in their teens and who have reported on sexual orientation (Li, Kung, & Hines, 2017). While there are no reports directly linking fetal testosterone to sexual orientation, an early ALSPAC study (Hines, Golombok, Rust, Johnston, & Golding, 2002) found that sex hormone binding globulin, a correlate of fetal testosterone, was related to sex-typed behavior in girls, but not boys, at age 3.5; and 15 years later, the Li et al. study showed that behavior measured in a sample of children drawn from the same population at ages 3.5 and 4.7 years was predictive of sexual orientation at age 15 years in both males and females. These findings are consistent with Breedlove’s conclusion that fetal testosterone plays a role in female, but not male, sexual orientation. Nevertheless, direct evidence confirming this conclusion remains forthcoming.

With respect to the use of biomarkers as proxies for fetal androgen exposure, there is a wealth of literature linking 2nd to 4th finger–length ratio (2D:4D) to various behaviors that also show a sex difference, including sexual orientation. Before commenting on the merits of those studies, or a recent conclusive meta-analysis (Grimbos, Dawood, Burriss, Zucker, & Puts, 2010), however, the reliability of the biomarker itself as a proxy for fetal androgen exposure bears scrutiny. Despite thousands of studies assuming fetal androgen effects based on divergent 2D:4D measurements, there has been no clear or consistent evidence directly linking sexual dimorphism in 2D:4D to variance in fetal androgen exposure. Indirect evidence is not sufficient. A fundamental principle of the scientific process warns against the interpretation of a causal relationship between variables which have only been shown to covary. Van Hemmen et al. (2017) make this point in reporting on digit ratio in women with CAIS, where androgen action is impaired despite a male karyotype. Though Van Hemmen et al. found that proband-control 2D:4D measurements varied in a pattern consistent with a hormonal influences interpretation, closer inspection of within group variance suggested that fetal androgen alone could not explain the observations. They explicitly cautioned against the use of digit ratio as a dependable proxy and suggested that non-androgenic (e.g., sex chromosome) factors are also needed to establish the male-typical phenotype.

Reliability is the primary problem with evidence directly linking fetal androgen exposure to variance in 2D:4D. There is the early Manning, Bundred, Newton, and Flanagan (2003) report which suggested that 2D:4D finger–length ratios covaried meaningfully with a polymorphic repeat (CAG) sequence in the gene coding for androgen receptors in men; however, two subsequent and larger studies failed to find the same effect (Hampson & Sankar, 2012; Hurd, Vaillancourt, & Dinsdale, 2011). Manning et al. have never been replicated. Then, there are three further studies where a number of different hormones were measured at various points in pregnancy, including testosterone, estrogen, DHEA, and insulin-like factor 3 (NSILF3) among others (Lutchmaya, Baron-Cohen, Raggatt, Knickmeyer, & Manning, 2004; Mitsui et al., 2015, 2016). One of the three studies found a negative relationship between INSL3 and 2D:4D in a sample of 135 males (Mitsui et al., 2015), but not in females; another report described a negative relationship with DHEA, but again in males only (*N* = 135; Mitsui et al., 2016). Though the third study found a negative relationship between a ratio of fetal testosterone to fetal estrogen (FT/FE) and 2D:4D in a combined sample of males and females (*N* = 33), it is worth noting that this group of participants did not show the sex difference in 2D:4D which underpins the hypothesis driving such investigations (Lutchmaya et al., 2004).

Reliability is also a problem in studies of 2D:4D in girls with CAH, who would have been exposed to high levels of testosterone beginning early in gestation (Brown, Hines, Fane, & Breedlove, 2002; Buck, Williams, Hughes, & Acerini, 2003; Ökten, Kalyoncu, & Yariş, 2002; Rivas et al., 2014). Though Brown et al. found that girls with CAH had lower 2D:4D than unaffected girls, their sample size was small (*N* = 13 girls with CAH), making it difficult to generalize to much larger normative populations. Ökten et al. also found that girls with CAH had lower 2D:4D than control girls; however, they also had a small sample (9 boys and 17 girls) and included two patients with non-classical CAH (i.e., onset in later childhood), who would not have been exposed to high levels of androgen prenatally (note that it was not stated whether these were male or female participants). Only nine children with CAH (males/females) in their cohort were diagnosed in the neonatal period, suggesting that the 17 participants who were diagnosed later may have had a milder form of the condition. Similarly, in a study of Brazilian children with CAH, Rivas et al. found masculinized 2D:4D in female patients (*N* = 31). Once again, however, it was noted that none of these participants had received hormone replacement therapy prior to the study (*M* age = 10.7 years), suggesting that they probably had a milder form of the condition (subtypes of CAH were not stated). The issue of disease severity in CAH is important in light of evidence for a dose–response relationship between fetal androgen exposure and masculinized behavioral traits (Nordenstrom, Servin, Bohlin, Larsson, & Wedell, 2002). Furthermore, and in contrast to the three studies above, a fourth study included a large sample of 66 girls with CAH, all of whom were diagnosed with the classical form in the early neonatal period (Buck et al., 2003). Though the expected sex difference in 2D:4D was found for controls (*N* = 69 females, *N* = 77 males), no significant difference was found between girls with and without CAH.

Finally, several studies have employed a hormone transfer paradigm, where comparisons are made between co-twins from same-sex dizygotic (DZ) twin pairs and opposite-sex DZ twin pairs, to investigate potential links between fetal testosterone exposure and 2D:4D (Cohen-Bendahan, 2005; Medland, Loehlin, & Martin, 2008; van Anders, Vernon, & Wilbur, 2006; Voracek & Dressler, 2007). While the first study did not find any effects (Cohen-Bendahan, 2005), two subsequent studies did find evidence linking fetal androgen to variance in 2D:4D (van Anders et al., 2006; Voracek & Dressler, 2007). Given the conflicting evidence, and the fact that the two studies reporting effects employed very small samples (ranging between eight and ten twin pairs), Medland et al. conducted a large-scale investigation including 118 pairs of same-sex female DZ twins and 106 opposite-sex DZ twins. Despite the exceptionally large sample size, they did not find evidence that fetal hormone transfer between opposite-sex twins affected 2D:4D measured in adolescence. Taken together, these studies suggest that either hormone transfer does not occur or that fetal androgen exposure is not directly related to variance in 2D:4D. It is possible that positive findings for the putative link are tapping latent effects of heritability in digit ratio (Gobrogge, Breedlove, & Klump, 2008; Medland & Loehlin, 2008; Paul, Kato, Cherkas, Andrew, & Spector, 2006; Voracek & Dressler, 2007). In sum, trying to find reliable evidence directly linking fetal androgen to 2D:4D finger–length ratio is like searching for the Higgs Boson (NB: Scientists most definitely have found the Higgs Boson, but it wasn’t what they were looking for!).

And so, in consideration of the lack of clear support for 2D:4D finger–length ratio as primarily dependent on fetal androgen exposure, interpretation of the vast array of findings linking the marker to sexual orientation (or other sexually dimorphic outcomes) seems a near impossible task. There are plenty of studies finding directly conflicting results (e.g., Van Honk et al., 2011; Voracek & Dressler, 2006), others find effects in different hands (e.g., Atkinson, Smulders, & Wallenberg, 2017; Voracek, Pietschnig, Nader, & Stieger, 2011), and still others find variable effects for men versus women (e.g., Voracek et al., 2011; Wallien, Zucker, Steensma, & Cohen-Kettenis, 2008). Though various meta-analyses may take such factors into consideration (e.g., Grimbos et al., 2010), conclusions relying on 2D:4D as a proxy for fetal androgen exposure are simply based on a questionable premise. Nevertheless, Breedlove’s conclusion, in large part based on findings from the Grimbos et al. meta-analysis, that fetal androgen exposure relates to sexual orientation in females, but not males, is probably at least partially true. In support of this conclusion, Li et al. (2017) found a link between childhood sex-typed behavior and later sexual orientation in both sexes, while the link between sex-typed behavior and fetal testosterone levels was found for girls only (Hines et al., 2002). Given the comparatively robust and consistent evidence from longitudinal studies and studies of outcomes in women with CAH and CAIS, my view is that we can safely assume a role for early androgens in the development of sexual attractions in women. Selective findings from the 2D:4D literature do not strengthen that argument.

## Mini-Puberty: Effects of Perinatal Androgen Exposure

As Breedlove concluded, explaining homosexuality in men is a difficult task. By now, the majority of scientists studying the topic likely agree that homosexuality is definitely not a choice and probably not due to socioenvironmental factors. At the same time, there appear to be no physical indicators of disrupted fetal sexual differentiation in homosexual men that would fit with the basic premise of the hormone theory of sex development. However, it is possible that alterations in the androgen surge that occurs in the early postnatal period, also called mini-puberty, could have effects that are not immediately or physically obvious. Based on the finding that penile growth in the first three months of life correlates with a concomitant surge in serum testosterone levels (Boas et al., 2006), Pasterski et al. (2015) considered the possibility that penile growth may act as a proxy for neonatal androgen exposure and that change measurements may be related to later neurobehavioral outcomes. In a longitudinal study of 81 typically developing boys, we found that the strength of the early postnatal androgen surge, from birth to approximately three months of age, predicted masculine behavior at 4 years old. By controlling for effects of prenatal androgen exposure using measurements of penile length and anogenital distance (AGD; sexually dimorphic and roughly twice as long in males compared to females) at birth, we showed that penile growth in the first three months of life, but not thereafter, accounted for significant variance in later sex-typed behavior. In the overall regression analysis, which controlled for various factors, penile length at birth was not related to sex-typed behavior. This suggests that disruption to male mini-puberty could have implications for future sex-related outcomes that are masked by a typical appearance at birth. Further, this provides support for the hypothesis that early (postnatal) hormone exposure influences aspects of sex-typed development in men, in a similar fashion to prenatal hormone exposure that is presumed to affect women.

Though an ever-growing body of literature aims to elucidate mechanisms underlying human sexual orientation, there is potential for the “wealth” of knowledge to obfuscate genuine discoveries. Sifting through mountains of research that may or may not seem compatible in order to integrate evidence relevant to particular theoretical frameworks is a difficult task. However, if we are to understand the true nature of increasingly complex human experiences, we must be willing to modify our interpretations of scientific findings with an open mind aimed at collective intellectual growth. I agree with parts of Breedlove’s interpretation, but not others. Hopefully, perspectives presented in this Commentary can be integrated into new thinking about the original article.
